# Human Umbilical Cord Mesenchymal Stem Cells Ameliorate Cognitive Decline by Restoring Senescent Microglial Function via NF‐κB‐SREBP1 Pathway Inhibition

**DOI:** 10.1111/acel.70259

**Published:** 2025-10-13

**Authors:** Aihong Liang, Li Zhang, Jing Peng, Yanan Li, Yunduo Zhou, Chao Yang, Jie Wang, Yizhong Yan, Hua Mei, Jun Zhu, Siqi Wang, Na Xiao, Yu Zhou, Lamei Cheng

**Affiliations:** ^1^ Institute of Reproductive and Stem Cell Engineering, Xiangya School of Basic Medical Science Central South University Changsha China; ^2^ National Engineering Research Center of Human Stem Cells Changsha China; ^3^ NHC Key Laboratory of Human Stem Cell and Reproductive Engineering, Xiangya School of Basic Medical Science Central South University Changsha China; ^4^ Hunan Guangxiu Hi‐Tech Life Technology Co. Ltd Changsha China; ^5^ Guangxiu Hospital Hunan Normal University Changsha China; ^6^ Department of Neurosurgery The Second Xiangya Hospital of Central South University Changsha China

**Keywords:** aging, hUC‐MSCs, lipid droplets, microglia, neuron

## Abstract

Aging is a major risk factor for neurodegenerative diseases, yet the role of senescent microglia in age‐related cognitive dysfunction remains incompletely understood. Human umbilical cord‐derived mesenchymal stem cells (hUC‐MSCs) have been extensively studied for their significant potential in anti‐aging. In this study, we demonstrated that hUC‐MSCs ameliorate age‐related cognitive decline and downregulate senescence‐associated markers in the aged hippocampus. Furthermore, co‐culture experiments showed that senescent microglia exacerbate neuronal senescence and neuroinflammation, while also suppressing the apoptosis of senescent neurons. These findings suggested that senescent microglia contribute to age‐related cognitive decline by exacerbating neuronal damage and impairing senescent neurons' clearance. We also elucidated a novel mechanism by which hUC‐MSCs alleviate age‐related cognitive decline by targeting senescent microglia. Specifically, we showed that hUC‐MSCs reduce senescence‐associated markers, decrease lipid droplet accumulation, and restore phagocytic function in senescent microglia through the inhibition of the NF‐κB‐SREBP1 pathway. This pathway modulation attenuates neuronal damage and promotes the apoptosis of senescent neurons, facilitating the clearance of damaged neurons. These findings highlight the therapeutic potential of hUC‐MSCs in age‐related neurodegenerative disorders.

## Graphical abstract

Human umbilical cord‐derived mesenchymal stem cells (hUC‐MSCs) ameliorate age‐related cognitive decline by targeting senescent microglia. They attenuate microglial senescence, reduce LD accumulation, and inhibit the NF‐κB‐SREBP1 signaling pathway, ultimately mitigating neuroinflammation and neuronal damage.

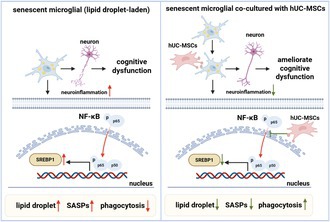

## 1 Introduction

A hallmark of cellular senescence is the accumulation of intracellular lipid droplets (Mutlu et al. 2021). In the aging brain, these lipid droplets primarily accumulate in glial cells, especially microglia (Marschallinger et al. 2020). Microglia, the resident immune cells of the central nervous system (CNS), play a critical role in maintaining neural homeostasis by monitoring the CNS microenvironment, remodeling and pruning synapses, and clearing cellular debris through phagocytosis (Vecchiarelli and Tremblay 2023). Recent studies have identified a distinct subpopulation of microglia termed lipid droplet‐accumulating microglia (LDAM), which exhibit a unique phenotype characterized by metabolic reprogramming, elevated oxidative stress, and heightened pro‐inflammatory responses (Marschallinger et al. 2020). These alterations disrupt microglial homeostasis, impair their ability to clear amyloid‐beta plaques and tau protein aggregates, and contribute to the progression of neurodegenerative diseases such as Alzheimer's disease (AD) (Uddin and Lim 2022). However, the role of senescent, lipid droplet‐laden microglia in cognitive decline remains poorly understood.

Lipid droplets (LDs) are lipid‐rich organelles enveloped by a phospholipid monolayer, primarily composed of triglycerides and cholesterol esters. They are present in diverse cell types, including microglia and neurons (Conte et al. 2021). Their formation occurs in the rough endoplasmic reticulum through the concerted action of various lipid‐synthesizing enzymes (Walther et al. 2017). Under physiological conditions, the homeostasis of intracellular LDs is tightly regulated by the balance among lipid synthases, lipases, and autophagic degradation pathways such as lipophagy, thereby supporting cellular energy metabolism and lipid homeostasis (Mathiowetz and Olzmann 2024). However, under pathological conditions, this balance is disrupted, leading to lipid droplet accumulation and subsequent cellular dysfunction. Accumulating evidence shows that LD content in microglia and neurons increases with age (Marschallinger et al. 2020; Conte et al. 2021). In neurons, age‐related LD accumulation is closely associated with neuroinflammation and may represent an early step in neurodegeneration (Conte et al. 2021). In microglia, LD enrichment is linked with a pro‐inflammatory phenotype and exhibits reduced phagocytic capacity toward cellular debris and protein aggregates (Arbaizar‐Rovirosa et al. 2022). In the context of AD, LDAM promote abnormal amyloid accumulation, exacerbate neuroinflammation, and intensify neuronal damage, thereby worsening disease pathology (Lee et al. 2023). Thus, elucidating the impact of lipid droplet accumulation on senescent microglia function and understanding the contribution of LDAM to neuroinflammation and neurodegeneration may provide novel therapeutic targets for addressing age‐related cognitive decline and neurodegenerative diseases.

hUC‐MSCs exhibit robust proliferative capacity, multipotent differentiation potential, low immunogenicity, and potent paracrine activity, making them highly promising for regenerative medicine (Spees et al. 2016). Their immunomodulatory, tissue repair, and neural regeneration potential have attracted considerable attention in neuroscience (Song et al. 2020; Giovannelli et al. 2023). Accumulating evidence confirms that MSCs exert their regulatory effects primarily through paracrine secretion of soluble factors (e.g., cytokines, growth factors) and extracellular vesicles (Guo et al. 2020). For instance, MSCs secrete anti‐inflammatory cytokines such as interleukin‐10 (IL‐10) and transforming growth factor‐β (TGF‐β), which modulate immune responses, improve the neuroimmune microenvironment, and potentially mitigate neurodegenerative changes (Shi et al. 2018; Asgari Taei et al. 2022). In addition, hUC‐MSCs have been reported to enhance synaptic plasticity in senescent neurons and promote endogenous neurogenesis (Cao et al. 2017; Hoang et al. 2022). Notably, exosomes secreted by hUC‐MSCs also contribute to neural repair. For example, hUC‐MSC‐derived exosomes have been shown to improve cognitive dysfunction in Parkinson's disease (PD) models by enhancing neuronal autophagy (Chen et al. 2020). Moreover, these exosomes have been shown to alleviate inflammatory pain and improve neurological outcomes following ischemic stroke (Che et al. 2023). Furthermore, extracellular vesicles derived from MSCs have been reported to attenuate AD pathogenesis (Cone et al. 2021), reduce endothelial cell senescence, and promote vascular regeneration (Xiao et al. 2021). Nevertheless, it remains unclear whether hUC‐MSCs can modulate the lipid metabolism of senescent microglia, thereby enhancing their phagocytic capacity, mitigating neuronal damage, and improving cognitive dysfunction in the aging brain.

In this study, we utilized both a naturally aging mouse model and an H_2_O_2_‐induced senescent microglia model to explore the potential mechanism through which hUC‐MSCs might exert neuroprotective effects. Our findings aim to provide a theoretical foundation for the clinical application of hUC‐MSCs in the treatment of aging‐associated cognitive impairment and neurodegenerative diseases.

## 2 Materials and Methods

### 2.1 Mice

All experimental procedures were approved by the Animal Ethics Committee of Central South University (permit number: CSU‐2023‐0480). Twelve male C57BL/6 mice (8 weeks, 20.25 ± 0.6 g) were procured from Hunan Slaike Jingda Laboratory Animal Co. LTD (permit number: SCXK (Xiang) 2020‐0019). Mice were raised with free access to food and water in a temperature‐controlled room (23°C ± 2°C) with a normal dark–light (12 h: 12 h) cycle. Until reaching 18 months of age, the mice were fed a standard diet and then randomly allocated into two groups: the old‐vehicle group (*n* = 6) and the old‐MSC group (*n* = 6). The old‐MSC group was intravenously injected with hUC‐MSCs (1 × 10^6^ cells/200 μL/mouse) via the caudal vein once a week for four consecutive weeks. Meanwhile, the old‐vehicle group was injected with hUC‐MSCs vehicle (200 μL/mouse) in the same way. Additionally, six male C57BL/6 mice (8 weeks, 20.19 ± 0.8 g) were obtained from the same supplier and served as the young group. The young group received a standard diet without any intervention.

### 2.2 Cell Culture

BV2 cells (a mouse microglia cell line) and HT22 cells (a mouse neuron cell line) were purchased from Procell Life Science & Technology Co. Ltd. and cultured in Dulbecco's Modified Eagle Medium (DMEM, Gibco) supplemented with 10% Fetal Bovine Serum (FBS, Invitrogen) in an atmosphere of 5% CO_2_ at 37°C.

hUC‐MSCs were supplied by the National Engineering Research Center of Human Stem Cells and the Guangxiu Tech Biotechnology Co. The preparation was carried out in a Good Manufacturing Practice (GMP) laboratory for medical products, as previously reported. Briefly, the master cell bank at passage 1 and the working cell bank at passage 4 were identified by the criteria suggested by the International Society for Cellular Therapy (ISCT): (1) plastic adherent in standard culture conditions using tissue culture flasks; (2) ≥ 95% of the MSC population express CD105, CD73, and CD90 and lack expression of (≤ 2% positive) CD45, CD34, CD11b, CD19, and HLA‐DR as measured by flow cytometry (BD Accuri C6); (3) differentiation potential to osteoblasts, adipocytes, and chondroblasts under standard in vitro differentiating conditions. Moreover, each batch of the master cell bank and the working cell bank was tested for sterility, human virus, G‐banding karyotype analysis, tumorigenicity, immunomodulatory capacity, telomere length, and telomerase activity. In preparation for infusion, frozen UMSCs at passage 4 were quickly thawed, washed, and suspended in normal saline supplemented with human serum albumin. For each MSC product, cell counts and viability were examined using trypan blue staining by a cell counter (Countstar) as product release criteria (Cheng et al. 2022).

hUC‐MSCs possess inherent immunosuppressive properties and low immunogenicity, which enable them to evade a robust host immune response following transplantation into mice (Naji et al. 2019). Our previous work has also demonstrated that hUC‐MSC transplantation in mice does not induce an abnormal immune response; instead, it suppresses excessive immune response, supporting their safety and efficacy in cross‐species applications (Peng et al. 2025).

### 2.3 Senescent Cell Model and Treatment

To establish a cellular model of senescent microglia and neurons, BV2 and HT22 cells were plated in 24‐well plates for 24 h and subsequently treated with hydrogen peroxide (H_2_O_2_, Merck) at concentrations of 50, 80, 100, 120, and 150 μM for an additional 24 h. Then, the optimal concentration for inducing cellular senescence was determined using BODIPY staining and Senescence‐Associated β‐galactosidase staining. After the senescent cell models were successfully constructed, senescent microglia were co‐cultured with hUC‐MSCs by using a Transwell system (0.4 μm pore) for 24 h. Meanwhile, senescent microglia were treated with 10 μM JSH‐23 (NF‐κB inhibitor) for 24 h to evaluate the role of NF‐κB in lipid droplet‐accumulating microglia.

To investigate whether hUC‐MSCs alleviate senescent microglia‐induced neuronal damage by improving senescent microglial function, we divided the HT22 cells into four groups: (1) ctrl group: HT22 cells without any treatment; (2) H_2_O_2_ group: senescent neurons group: cells treated with 80 μM H_2_O_2_ for 24 h; (3) B‐H group: senescent microglia–neuron co‐culture group (BV2‐H_2_O_2_/HT22‐H_2_O_2_): BV2 cells were treated with H_2_O_2_ for 24 h, then co‐cultured with H_2_O_2_‐induced senescent HT22 neurons for another 24 h; (4) B‐M group: hUC‐MSC‐preconditioned senescent microglia–neuron co‐culture group (BV2‐H_2_O_2_‐MSC/HT22‐H_2_O_2_): senescent BV2 cells (H_2_O_2_‐treated) were co‐cultured with hUC‐MSCs for 24 h, then co‐cultured with senescent HT22 neurons for an additional 24 h.

### 2.4 T‐Maze Test

The T‐maze test was respectively performed on mice before and one week after the final injection of hUC‐MSCs. The T‐maze apparatus consists of two goal arms (22 × 5 × 5 cm) and a stem that is perpendicular to them, measuring 45 cm in length, with the same width and height as the goal arms. The approach alley contains a 5 × 5 cm starting box. At the start of the experiment, the mouse was placed in the stem. The gate was then opened, allowing the mouse to leave the main arm and enter one of the goal arms (defined as all four limbs crossing into the arm). After each entry, the mouse was immediately returned to the main arm and kept there for 5 s. This process was repeated 9 times, with the number of entries into each arm being recorded. The success rate was calculated by dividing the number of alternating choices within the same experimental session by the total number of choices made.

### 2.5 Novel Object Recognition Test

The novel object recognition test (NOR) test was respectively performed on mice before and one week after the final injection of hUC‐MSCs. The NOR apparatus consists of an open‐field box (25 × 25 × 30 cm), two identical objects, and a new object, situated in a quiet room. The NOR test consists of two steps: the familiarization phase and the test phase. During the 5‐min familiarization phase, two identical objects are placed in two non‐opposite corners of the box, equidistant from the periphery. The animal is then placed at the center of the box to explore freely for 5 min. After a 60‐min intertrial interval, the test phase begins, where one of the two identical objects is replaced with a novel object. Each mouse performs the test individually, and the box and objects are cleaned with 75% alcohol after each test. Exploration behavior is defined as the animal turning its nose toward the objects at a distance of ≤ 3 cm or touching them with the nose or forepaw, while passing by, climbing, or sitting on the object is not included. The recognition index (RI) is calculated as the percentage of time spent exploring the novel object over the total time spent exploring both objects.

### 2.6 Senescence‐Associated β‐Galactosidase Staining

The Senescence‐Associated β‐galactosidase (SA‐β‐gal) staining kit (Beyotime) was used to detect SA‐β‐gal activity in brain tissues and BV2 or HT22 cells. Brain tissues frozen sections and cells were fixed for 15 min with fixative solution at room temperature, followed by incubation in staining solution overnight at 37°C according to the manufacturer's protocol. Images were captured by Nikon ECLIPSE TS100 microscopy, and the number of SA‐β‐gal‐positive cells was quantified using ImageJ software.

### 2.7 Immunofluorescence

Tissue sections were fixed in 4% paraformaldehyde and embedded in paraffin. 5 μm thick sections of brain tissue were baked in an oven at 65°C for 2 h, deparaffinized with xylene, dehydrated with gradient ethanol, and repaired with citrate repair solution for antigen. Next, peroxidase activity was blocked with 3% H_2_O_2_ and non‐specific antigens were blocked with 5% donkey serum for 1 h. Then sections were incubated overnight at 4°C with guinea pig anti‐PLIN2 antibody (Fitzgerald LLC, 1:200) and rabbit anti‐IBA1 antibody (Wako, 1:100). After washing with PBS three times, tissue sections were incubated with Alexa Fluor 488‐Donkey Anti‐Rat IgG (H + L) (Abcam, 1:500) and Alexa Fluor 647‐Goat anti‐Guinea Pig IgG (H + L) (ThermoFisher, 1:500) for 1 h. Nuclei were counterstained with DAPI (Sigma, 1:1000) at room temperature for 3 min. Images were captured by ZEISS Axioscope 5 fluorescence microscope.

### 2.8 Transmission Electron Microscopy

After euthanasia, the mice were sequentially perfused intracardially with normal saline and 4% paraformaldehyde (PFA). The hippocampal tissues were then immersed in an electron microscopy fixative solution. The tissues were embedded in resin, and ultrathin sections were prepared using a Leica EM UC7 ultramicrotome. The sections were stained with heavy metals (lead and uranium) and imaged with a Hitachi HT7700 transmission electron microscope at 80 kV.

### 2.9 BODIPY Staining

BODIPY is a neutral lipid dye. BV2 cells were seeded in a 24‐well plate at 2 × 10^4^ peer well. Following H_2_O_2_ treatment and co‐culture with hUC‐MSCs, the cells were fixed in 4% PFA for 30 min at room temperature. After washing with DPBS, the cells were incubated in DMEM containing 2 μM BODIPY 493/503 (ThermoFisher Scientific) at 37°C for 10 min. Subsequently, the nuclei were counterstained with DAPI (Sigma‐Aldrich) at room temperature for 3 min. Fluorescent images were captured using a ZEISS Axioscope5 fluorescence microscope, and the mean fluorescence intensity of BODIPY was analyzed using ImageJ software.

### 2.10 Phagocytic Assay

BV2 cells were seeded in a 24‐well plate at 2 × 10^4^ per well. After H_2_O_2_ treatment and hUC‐MSCs co‐culture, cells were incubated with 100 μg/mL pHRodo Green Zymosan Bioparticles (ThermoFisher Scientific) in DMEM at 37°C for 15 min. The nuclei were counterstained with Hoechst (Beyotime) at 37°C for 5 min. Fluorescent images were acquired using a ZEISS Axioscope5 fluorescence microscope, and the mean fluorescence intensity of zymosan was analyzed using ImageJ software.

### 2.11 Transcriptome Sequencing and Bioinformatics Analysis

RNA sequencing of hippocampal tissues was performed by Beijing Genomics Institute (BGI). Briefly, RNA samples were denatured, and mRNA were enriched using oligo (dT)‐attached magnetic beads. After fragmentation, cDNA synthesis was performed, followed by end‐repair, A‐tailing, and PCR amplification. The final DNA nanoballs (DNBs) were loaded into patterned nanoarrays and sequenced using combinatorial Probe‐Anchor Synthesis (cPAS). The sequencing data was filtered using SOAPnuke, and gene expression levels were calculated with RSEM (v1.3.1). Differential expression analysis was performed using DESeq2 (v1.4.5), and KEGG enrichment analysis was conducted using Phyper based on the Hypergeometric test, with a significance threshold of *p* value ≤ 0.05.

### 2.12 Western Blot

Hippocampal tissues and BV2 cells were homogenized in lysis buffer (RIPA [Beyotime]: PMSF [BOSTER] 100:1) on ice for 30 min, followed by centrifugation at 12000 rpm for 20 min at 4°C. Protein concentration was measured by BCA Protein Assay Kit (Beyotime). Proteins were denatured by boiling at 100°C for 10 min and then separated by 10% or 12% SDS‐PAGE and transferred onto polyvinylidene fluoride (PVDF, Millipore) membranes. Membranes were blocked with 5% skim milk powder in TBST for 2 h, washed with TBST, and incubated overnight at 4°C with primary antibodies. After washing with TBST, membranes were further incubated with HRP‐conjugated secondary antibodies for 2 h at room temperature. Super Signal Enhancer Chemiluminescent (ECL plus, Nature biosciences) was used to detect chemiluminescence with the Fusion FX5 Chemiluminescence Imaging System.

The following primary and secondary antibodies were used: rabbit anti‐p16 (CST, 1:1000), rabbit anti‐p21 (Abcam, 1:1000), rabbit anti‐γ‐H2A.X (CST, 1:1000), guinea pig anti‐PLIN2 (Fitzgerald LLC, 1:1000), rabbit anti‐SREBP1 (Abcam, 1:1000), rabbit anti‐p65 (CST, 1:1000), rabbit anti‐p‐p65 (CST, 1:1000), and rabbit anti‐β‐actin (Abcam, 1:3000), goat anti‐mouse IgG H&L (Abcam, 1:1000), goat anti‐rabbit IgG H&L (Abcam, 1:1000).

### 2.13 Quantitative Real‐Time PCR

Total RNA from hippocampal tissues and BV2 or HT22 cells was extracted using the TRIzol reagent (Apex BIO). cDNA synthesis was performed with the Go Script Reverse Transcription system (Promega). Real‐time PCR was performed with SYBR GREEN 480 (Roche). β‐actin was used as the reference gene, and gene expression levels were calculated using the 2^−ΔΔ*Ct*^ method. The following primers were used: β‐actin forward primer (5′‐GTGACGTTGACATCCGTAAAGA‐3′) and reverse primer (5′‐GCCGGACTCATCGTACTCC‐3′); Cdkn2a (p16Ink4a) forward primer (5′‐CCCAACGCCCCGAACT‐3′) and reverse primer (5′‐GCAGAAGAGCTGCTACGTGAA‐3′); Cdkn1a (p21Cip1) forward primer (5′‐GTCAGGCTGGTCTGCCTCCG‐3′) and reverse primer (5′‐CGGTCCCGTGGACAGTGAGCAG‐3′); TNF‐α forward primer (5′‐GTCTGTATCCTTCTAACTTA‐3′) and reverse primer (5′‐TCTTGTGTTTCTGAGTAG‐3′); TGF‐β forward primer (5′‐CTGCAAGAGACTTCCATCCAG‐3′) and reverse primer (5′‐TGGTTTTCTCATAGATGGCG‐3′); IL‐6 forward primer (5′‐CTGCAAGAGACTTCCATCCAG‐3′) and reverse primer (5′‐AGTGGTATAGACAGGTCTGTTGG‐3′); Cxcl2 forward primer (5′‐CCAACCACCAGGCTACAGG‐3′) and reverse primer (5′‐GCGTCACACTCAAGCTCTG‐3′).

### 2.14 Statistical Analysis

Data were analyzed using GraphPad Prism 8.0 Software and presented as the mean ± standard deviation. Significant differences between groups were evaluated by one‐way ANOVA following normality and lognormality tests.

## 3 Results

### 3.1 hUC‐MSCs Ameliorate Cognitive Decline and Hippocampal Senescence in Aged Mice

**FIGURE 1:**
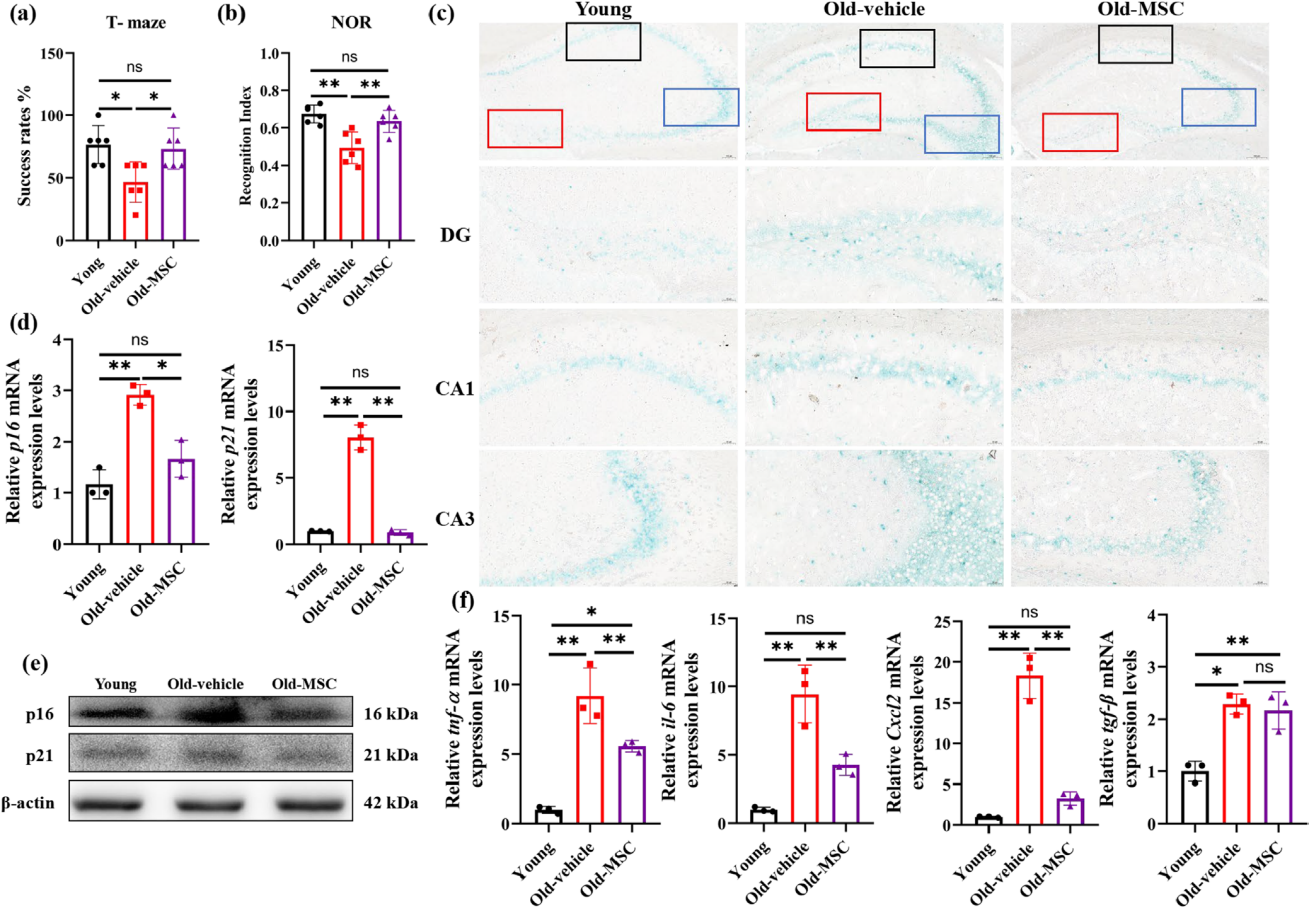
hUC‐MSCs ameliorate cognitive decline and hippocampal senescence in aged mice. (a) The spatial memory of young, aged mice with hUC‐MSCs vehicle or hUC‐MSCs were assessed in the T‐maze test. *n* = 6 per group. (b) The short memory of young, aged mice with hUC‐MSCs vehicle or hUC‐MSCs were assessed in the NOR test. *n* = 6 per group. (c) Representative image of SA‐β‐gal staining in mice brain. The red box refers to the dentate gyrus (DG) region, blank refers to cornu ammonis 1 (CA1) region and blue refers to cornu ammonis 3 (CA3) regions. Scale bar 100 μm. (d) qPCR analysis of *p16*, *p21* mRNA levels in the hippocampus of mice. *n* = 3 per group. (e) Hippocampus lysates were immunoblotted for p16, p21. *n* = 4 per group. (f) The mRNA levels of SASP in the hippocampus of mice. *n* = 3 per group. Data are presented as means ± SD. Statistical significance was determined by One‐way ANOVA, following normality and lognormality tests. ns, no significant, *p < 0.05, **p < 0.01.

To evaluate the therapeutic potential of hUC‐MSCs on the age‐related cognitive decline, hUC‐MSCs were injected intravenously into aged mice (Figure S1a). Cognitive assessments using the T‐maze and NOR tests revealed significant improvements following hUC‐MSCs treatment. Aged mice demonstrated impaired performance in the T‐maze test (46.67% ± 2.89%) compared to young controls (76.67% ± 3.82%; *p* < 0.05), with hUC‐MSCs treatment restoring performance to youthful levels (73.33% ± 4.16%; *p* < 0.01 vs. aged group) (Figure 1a). Consistent with these findings, the NOR test revealed a reduced recognition index in aged mice (0.45 ± 0.03 vs. young: 0.67 ± 0.05; *p* < 0.01), which was significantly improved by hUC‐MSCs administration (0.64 ± 0.04; *p* < 0.01 vs. aged) (Figure 1b). Notably, total exploration times remained comparable between old and hUC‐MSCs treatment groups, though both were reduced relative to young controls (Figure S1b). Furthermore, the elevated SA‐β‐gal activity was observed in the aged hippocampus. hUC‐MSCs administration effectively attenuated this senescence‐associated phenotype, significantly reducing SA‐β‐gal activity compared to the old group (Figure 1c, Figure S1c).

Consistent with the observed phenotypic changes, the mRNA expression levels of senescence‐associated markers *Cdkn2a* (*p16* ^*INK4a*^) and *Cdkn1a* (*p21* ^*WAF1*^) were significantly upregulated in the aged hippocampus compared to young controls, which were decreased after hUC‐MSCs administration (Figure 1d). Western blot analysis confirmed corresponding increases in p16^INK4a^ (1.24‐fold) and p21^WAF1^ (1.2‐fold) protein expression in aged mice, with significant reductions post‐treatment (Figure 1e, Figure S1d). Moreover, the mRNA levels of senescence‐associated secretory phenotype (SASP) markers, such as interleukin‐6 (*il‐6*), tumor necrosis factor‐α (*tnf‐α*), transforming growth factor‐β (*tgf‐β*), and *Cxcl2*, exhibited parallel changes in the aged hippocampus, all of which were attenuated by hUC‐MSCs treatment (Figure 1f). These findings demonstrate that hUC‐MSCs treatment not only rescues age‐related cognitive deficits but also systemically decreases the expression of senescence‐related markers, including cell cycle arrest and SASP in the aged hippocampus.

### 3.2 hUC‐MSCs Attenuate Age‐Related Microglial Activation and Reduce LD Accumulation in Hippocampus

**FIGURE 2:**
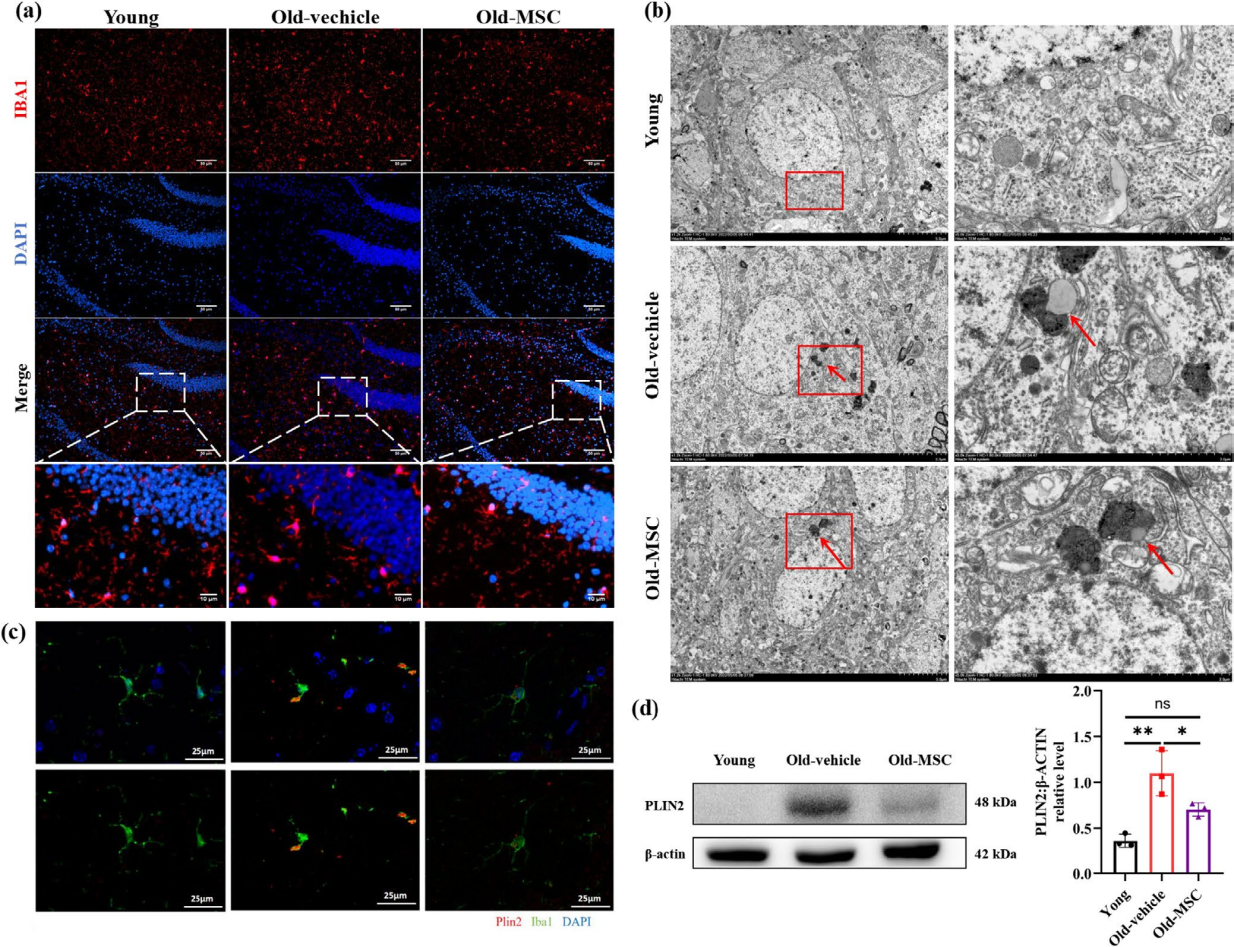
hUC‐MSCs attenuate age‐related microglial activation and reduce LD accumulation in hippocampus. (a) Representative image of microglia (IBA1 in red) in mice hippocampus. Scale bar 50 μm. (b) Representative image of TEM of microglia in mice hippocampus. Scale bar 5 μm. (c) Representative image of microglia (PLIN2 in red and IBA1 in green) in mice hippocampus. Scale bar 25 μm. (d) Hippocampus lysates were immunoblotted and quantified for PLIN2. *n* = 3 per group. Data are presented as means ± SD. Statistical significance was determined by One‐way ANOVA, following normality and lognormality tests. ns, no significant, *p < 0.05, **p < 0.01.

To investigate the effects of hUC‐MSCs on hippocampal microglial activation, we assessed microglial morphology using immunofluorescence. Results showed that the number of IBA1‐positive microglia was increased in the aged hippocampus compared to young controls, and these cells exhibited enlarged cell bodies and reduced branching, consistent with activated microglia (Figure 2a, Figure S2a,b). hUC‐MSCs transplantation decreased the number of microglia and restored ramified morphology (Figure 2a, Figure S2a,b). Transmission Electron Microscopy (TEM) ultrastructural analysis revealed that LDs were accumulated in the microglia of the aged hippocampus, which exhibited direct membrane contact with lysosomes (red arrow) (Figure 2b). hUC‐MSCs treatment significantly reduced LD size, with LDs fully encapsulated within lysosomal compartments (red arrow) (Figure 2b). The phospholipid monolayer of LDs is enriched with lipoproteins, among which perilipins (PLIN 1–5) are the most abundant. PLIN2 is widely present in non‐adipose cells and localizes to the LD surface, making it a universal marker for LDs (Conte et al. 2021). To specifically detect the content of LDs in microglia of the aged hippocampus, we performed immunofluorescence staining using PLIN2 (LD marker) and IBA1 (microglial marker). Consistent with TEM observations, PLIN2^+^ LDs were accumulated in IBA1^+^ microglia in the aged hippocampus, which was attenuated by hUC‐MSCs transplantation (Figure 2c). Western blot analysis confirmed the increase of the PLIN2 protein levels in the aged hippocampus, with hUC‐MSCs transplantation reducing the expression (Figure 2d). These results collectively demonstrate that hUC‐MSCs restore microglial quiescence in the aged hippocampus and enhance the lysosomal‐mediated lipid clearance.

### 3.3 hUC‐MSCs Attenuate H_2_O_2_ ‐Induced Microglial Senescence

**FIGURE 3:**
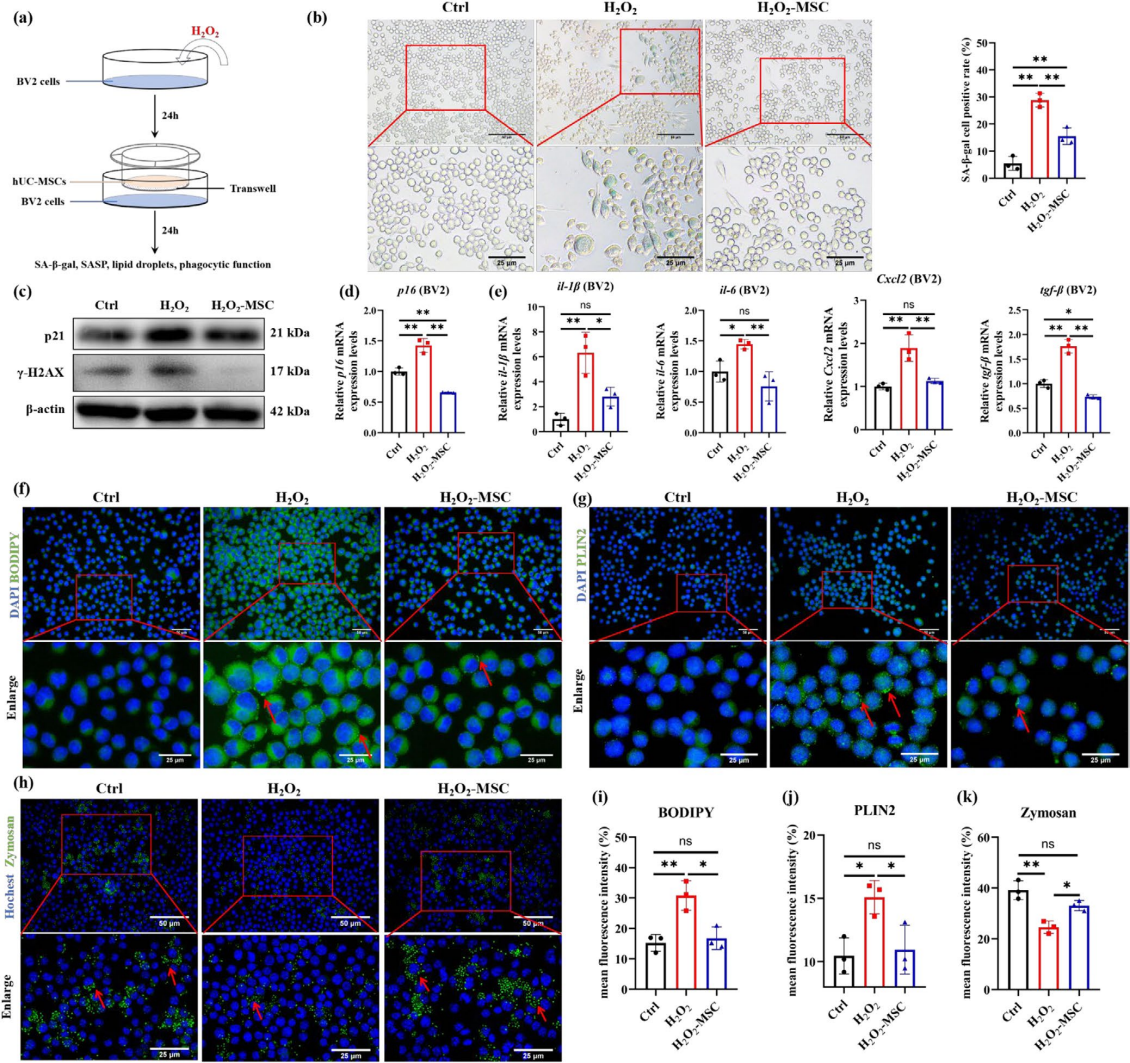
hUC‐MSCs attenuate H_2_O_2_‐induced microglial senescence, reduce LD accumulation and restore phagocytic function. (a) Schematics of BV2 cells treatment. (b) Representative image of SA‐β‐gal staining in BV2 cells and statistical analysis of the proportion of positive cells, *n* = 3 per group. (c) The protein levels of p21 and γ‐H2AX were determined by western blot and quantified using ImageJ software. *n* = 3 per group. (d‐e) *p16* and SASP mRNA levels were determined by qPCR and quantified using ImageJ software. *n* = 3 per group. (f) Representative image of BODIPY (green) in BV2 cells and (i) the mean fluorescence intensity of BODIPY was quantified by ImageJ software. *n* = 3 per group. (g) Representative image of PLIN2 (green) in BV2 cells and (j) the mean fluorescence intensity of PLIN2 was quantified by ImageJ software. *n* = 3 per group. (h) Representative image of zymosan (green) in BV2 cells and (k) the mean fluorescence intensity of zymosan was quantified by ImageJ software. *n* = 3 per group. Data are presented as means ± SD. Statistical significance was determined by One‐way ANOVA, following normality and lognormality tests. ns, no significant, *p < 0.05, **p < 0.01.

To investigate the anti‐senescence effect of hUC‐MSCs on microglia, we established an H_2_O_2_‐induced senescent microglia model in vitro. Dose–response analysis (50, 80, 100, 120, and 150 μM H_2_O_2_) revealed cell viability changes: 80–100 μM H_2_O_2_ induced cell proliferation, while ≥ 120 μM H_2_O_2_ caused cytotoxicity (Figure S3a). SA‐β‐gal activity revealed dose‐dependent increases from 80 to 100 μM (Figure S3b). Based on these results, 80 μM H_2_O_2_ was selected for subsequent senescence modeling (Figure 3a). Senescent BV2 cells were co‐cultured with hUC‐MSCs; a significant decrease in SA‐β‐gal activity was observed (Figure 3b). Western blot analysis demonstrated that hUC‐MSCs mediated the suppression of p21 and γ‐H2AX expression compared to senescence controls (Figure 3c, Figure S3c). hUC‐MSCs also downregulated *p16* expression and suppressed SASP expression such as *il‐1β, il‐6, tgf‐β*, and *Cxcl2* (Figure 3d,e). These in vitro results confirm that hUC‐MSCs exert potent anti‐senescence effects, effectively counteracting H_2_O_2_‐induced microglial senescence.

### 3.4 hUC‐MSCs Reduce LD Accumulation and Restore Phagocytic Function in H_2_O_2_ ‐Induced Senescent Microglia

To determine whether hUC‐MSCs restore senescent microglial function, we assessed LD content and phagocytic capacity in microglial senescence in vitro. BODIPY staining demonstrated H_2_O_2_ induced dose‐dependent LD accumulation (Figure S3d). Senescent microglia were co‐cultured with hUC‐MSCs; a significant decrease in BODIPY fluorescence intensity was observed (Figure 3f,i), indicating reduced LD accumulation. This finding was further confirmed by immunofluorescence and western blot analysis of PLIN2 expression (Figure 3g,j, Figure S3). Phagocytic assay revealed hUC‐MSCs significantly restored senescent microglial impaired phagocytic function (Figure 3h,k). These findings demonstrate that hUC‐MSCs reduce LD accumulation and improve phagocytic capacity in senescent microglia.

### 3.5 Senescent Microglia Exacerbate the Senescence of Aging Neurons

**FIGURE 4:**
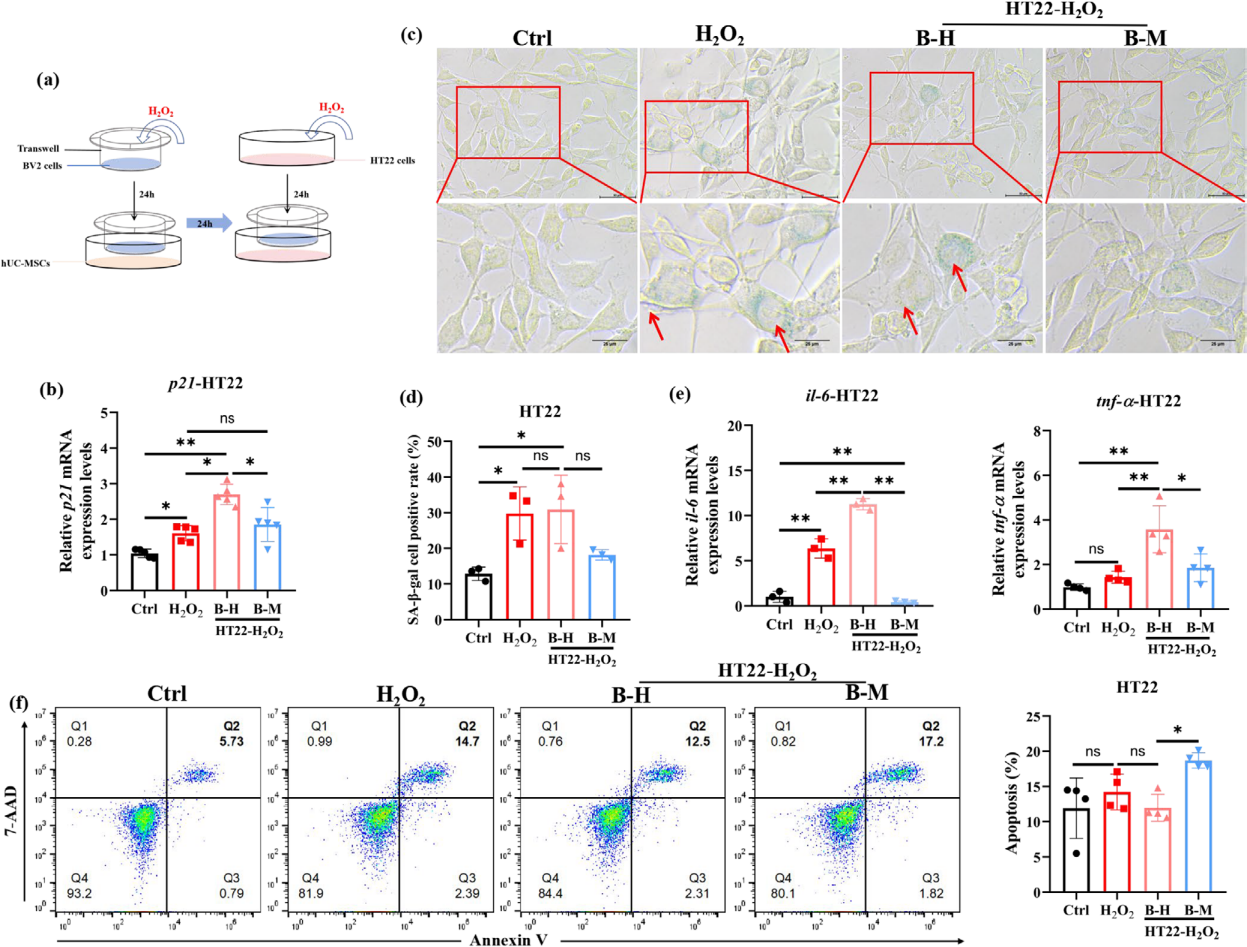
hUC‐MSCs alleviate senescent microglia‐induced damage to aging neurons. (a) Schematics of HT22 cells treatment. (b) qPCR analysis of *p21* mRNA levels (*n* = 5 per group). (c, d) Representative SA‐β‐gal staining image of HT22 cells and quantification of SA‐β‐gal positive HT22 cells (*n* = 3 per group). (e) qPCR analysis of *tnf‐α* (*n* = 4 per group) and *il‐6* (*n* = 3 per group) mRNA levels. (f) Representative flow cytometry plots and quantification of HT22 cell apoptosis (*n* = 3 per group). Data are presented as means ± SD. Statistical significance was determined by one‐way ANOVA, following normality and lognormality tests. ns = no significant, *p < 0.05, **p < 0.01. B‐H group: Senescent microglia–neuron co‐culture group (BV2‐H_2_O_2_/HT22‐H_2_O_2_): BV2 cells were treated with H_2_O_2_ for 24 h, then co‐cultured with H_2_O_2_‐induced senescent HT22 neurons for another 24 h. B‐M group: hUC‐MSC‐preconditioned senescent microglia–neuron co‐culture group (BV2‐H_2_O_2_‐MSC/HT22‐H_2_O_2_): Senescent BV2 cells (H_2_O_2_‐treated) were co‐cultured with hUC‐MSCs for 24 h, then co‐cultured with senescent HT22 neurons for an additional 24 h.

Microglia, as the resident immune cells of the central nervous system, are vital for maintaining neural homeostasis. To investigate the detrimental effects of senescent microglia on aging neurons, we established an H_2_O_2_‐induced neuronal senescence model in vitro and co‐cultured it with senescent microglia (Figure 4a). Senescent neurons exhibited a significant increase in *p21* mRNA expression, and this increase was further elevated after co‐culture with senescent microglia (Figure 4b). SA‐β‐gal activity revealed a significant increase in senescent neurons, which remained unaltered by co‐culture with senescent microglia (Figure 4c,d). Notably, co‐culture with senescent microglia markedly upregulated mRNA expression of the proinflammatory factors *tnf‐α* and *il‐6* in senescent neurons compared to the non‐co‐cultured group (Figure 4e). Furthermore, senescent neurons co‐cultured with senescent microglia (B‐H group) showed a trend toward reduced apoptosis compared to senescent neurons alone (H_2_O_2_ group): B‐H = 11.97% ± 1.198%, H_2_O_2_ = 14.21% ± 2.544%, *p* = 0.66 (Figure 4f). These findings suggest that senescent microglia can exacerbate neuronal senescence and neuroinflammation, thereby contributing to age‐related cognitive decline.

### 3.6 hUC‐MSCs Alleviate the Damage of Senescent Microglia on Aging Neurons

To assess whether hUC‐MSCs alleviate senescent microglia‐induced neuronal damage by improving senescent microglial function, we co‐cultured senescent neurons with senescent microglia that had been pre‐cocultured with hUC‐MSCs. Results showed that senescent neurons exhibited a significant decrease in *p21* mRNA expression (Figure 4b) and SA‐β‐gal activity after co‐culture with senescent microglia that had been pre‐cocultured with hUC‐MSCs (Figure 4c,d). Meanwhile, senescent microglia pre‐cocultured with hUC‐MSCs suppressed neuroinflammation by reducing the mRNA expression of *tnf‐α* and *il‐6* in senescent neurons (Figure 4e). Senescent neurons also exhibited an increase in apoptosis after co‐culture with senescent microglia that had been pre‐cocultured with hUC‐MSCs (B‐M: 18.70 ± 1.108 vs. B‐H: 11.97 ± 1.198; *p* = 0.02) (Figure 4f), indicating hUC‐MSC‐preconditioned senescent microglia promoted clearance of senescent neurons. These results indicate that hUC‐MSCs can alleviate senescent microglia‐induced neuronal damage by enhancing senescent microglial function to attenuate neuronal senescence, mitigate neuroinflammation, and promote senescent neuron clearance.

### 3.7 hUC‐MSCs Inhibit NF‐κB‐SREBP1 Pathway in Aged Hippocampus

**FIGURE 5:**
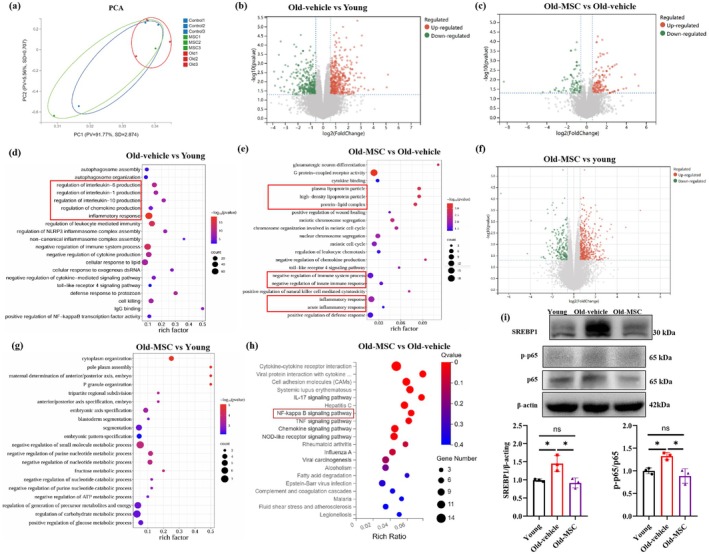
hUC‐MSCs inhibit NF‐κB‐SREBP1 pathway in aged hippocampus. (a) PCA demonstrates the variation in DEGs of three groups. (b) Volcano plots of DEGs in Old‐vehicle vs. young groups. (c) Volcano plots of DEGs in Old‐MSCs vs. Old‐vehicle groups. (d) GO enrichment analysis of DEGs in Old‐vehicle vs. young groups. (e) GO enrichment analysis of DEGs in Old‐MSCs vs. Old‐vehicle groups. (f) Volcano plots of DEGs in Old‐MSCs vs. young groups. (g) GO enrichment analysis of DEGs in Old‐MSCs vs. young groups. (h) Functional enrichment analysis of DEGs in Old‐MSCs vs. Old‐vehicle groups. (i) The protein levels of SREBP1, p65, and p‐p65 were determined by western blot and quantified by using ImageJ software. *n* = 3 per group. Data are presented as mean ± SD; statistical significance was determined by one‐way ANOVA, following normality and lognormality tests; ns, no significance, *p < 0.05.

To explore the anti‐aging mechanisms of hUC‐MSCs, we performed RNA sequencing on mouse hippocampus. As shown in Figure 5a, hUC‐MSCs‐treated aged hippocampal samples cluster closer to young samples in principal component analysis (PCA) space, while untreated aged samples are distinct from both groups, indicating partial restoration of the “young‐like” transcriptional profile. Meanwhile, a total of 760 differentially expressed genes (DEGs) were identified in the aged hippocampus compared to young controls, with 429 upregulated and 331 downregulated (Figure 5b). Comparing hUC‐MSC‐treated aged hippocampus (Old‐MSC group) with untreated aged hippocampus (Old‐vehicle group), 261 DEGs were identified, including 162 upregulated and 99 downregulated genes (Figure 5c). GO Biological Processes (BP) enrichment showed that DEGs between untreated aged and young groups (760 DEGs) are enriched in age‐related pathways such as inflammatory response (Figure 5d). Although only the 261 DEGs were identified between hUC‐MSCs‐treated and untreated aged groups, they were significantly enriched in senescence‐associated processes, including immune response, inflammatory response, and lipid metabolism (Figure 5e). Additionally, we also analyzed the DEGs between hUC‐MSCs‐treated aged and young groups, identifying 750 DEGs (518 upregulated, 232 downregulated, Figure 5f). These DEGs were enriched in structure‐related gene networks (e.g., embryonic axis patterning, cytoplasmic organization) and metabolic regulation (e.g., glucose metabolism) (Figure 5g). These findings indicate that hUC‐MSCs shift the aging hippocampus toward a healthier state by regulating senescence‐associated pathways (inflammation, lipid metabolism) but do not fully restore structural or metabolic gene networks. KEGG pathway enrichment analysis revealed that DEGs (Old‐MSC vs. Old‐vehicle) were enriched in cell signaling and inflammation‐related pathways, particularly the NF‐κB signaling pathway (Figure 5h). Evidence indicates that NF‐κB could promote the secretion of inflammatory factors and upregulate the expression of sterol regulatory element‐binding protein 1 (SREBP1) to facilitate the formation of LDs (Yan and Horng 2020). Consistent with this, we found the expression of SREBP1 was significantly increased in aged hippocampus and markedly reduced after hUC‐MSCs transplantation (Figure 5i). Meanwhile, hUC‐MSCs transplantation significantly reduced the p‐p65/p65 ratio in the aged hippocampus, suggesting suppression of NF‐κB pathway activation (Figure 5i). These data suggest that hUC‐MSCs ameliorate age‐related cognitive decline via inhibition of the NF‐κB‐SREBP1 pathway in the aged hippocampus.

### 3.8 JSH‐23 Reduces LD Accumulation, Enhances Phagocytic Function, and Attenuates H_2_O_2_ ‐Induced Senescence in Microglia

**FIGURE 6:**
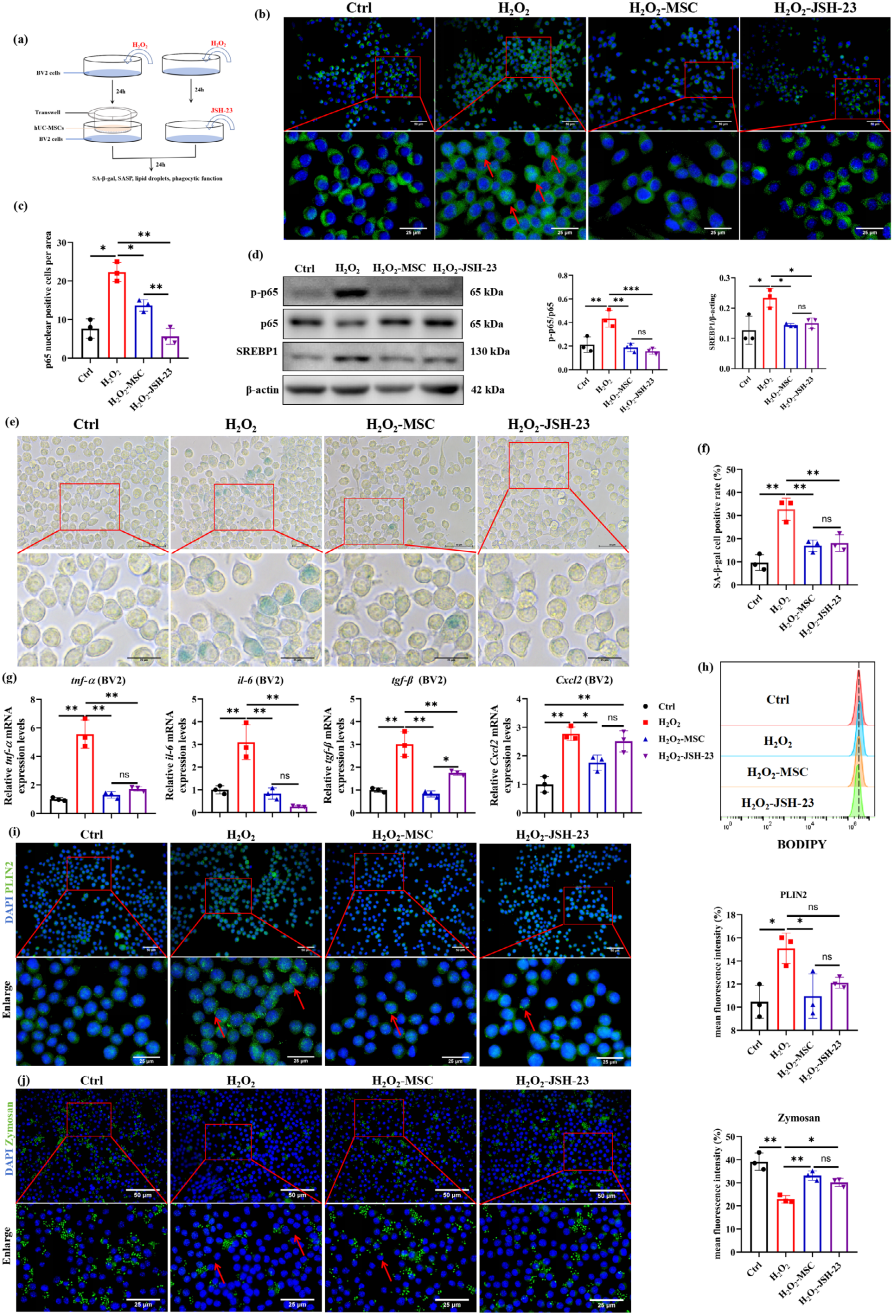
JSH‐23 reduces LD accumulation, enhances phagocytic function, and attenuates H_2_O_2_‐induced senescence in microglia. (a) Schematics of BV2 cells treatment. (b) Representative image of p65 (green) in BV2 cells and (c) statistical analysis of p65 nuclear‐positive cells. *n* = 3 per group. (d) The protein levels of p65, p‐p65 and SREBP1 were determined by western blot and quantified using ImageJ software. *n* = 3 per group. (e) Representative image of SA‐β‐gal in BV2 cells and (f) statistical analysis of positive cells. (g) The SASP mRNA levels were determined and quantified by qPCR. *n* = 3 per group. (h) Representative flow cytometry plots of BODIPY in BV2 cells. *n* = 3 per group. (i) Representative image of PLIN2 (green) in BV2 cells and mean fluorescence intensity quantified using ImageJ software. *n* = 3 per group. (j) Representative image of zymosan (green) in BV2 cells and mean fluorescence intensity quantified using ImageJ software. *n* = 3 per group. Data are presented as means±SD. Statistical significance was determined by One‐way ANOVA, following normality and lognormality tests. ns, no significant, *p < 0.05, **p < 0.01, ***p < 0.001.

To investigate whether hUC‐MSCs attenuate microglial senescence via inhibition of the NF‐κB‐SREBP1 pathway, we employed JSH‐23 to inhibit NF‐κB nuclear translocation in senescent microglia (Figure 6a). As shown in Figure 6b,c, JSH‐23 significantly inhibited NF‐κB nuclear translocation in senescent microglia, with a similar effect observed with hUC‐MSCs treatment. Western blot analysis further revealed that both JSH‐23 and hUC‐MSCs treatment significantly reduced the p‐p65/p65 ratio and SREBP1 protein level in senescent microglia (Figure 6d), indicating hUC‐MSCs inhibited the NF‐κB‐SREBP1 pathway. Consistent with the effects of hUC‐MSCs, JSH‐23 markedly reduced the proportion of SA‐β‐gal positive cell rate (Figure 6e,f) and suppressed the expression of SASP, including *il‐6*, *tnf‐α*, *tgf‐β*, and *Cxcl2* in senescent microglia (Figure 6g). These findings suggest that hUC‐MSCs attenuate microglial senescence through inhibition of the NF‐κB‐SREBP1 axis.

BODIPY staining, PLIN2 immunofluorescence, and oil red O staining demonstrated that JSH‐23 significantly reduced LD accumulation in senescent microglia (Figure 6h,i, Figure S4a–c), recapitulating the LD‐reducing effects observed with hUC‐MSC co‐culture. Additionally, JSH‐23 also significantly enhanced the phagocytic capacity in senescent microglia (Figure 6j). These data demonstrate that hUC‐MSCs reduce LD accumulation and enhance phagocytic capacity in senescent microglia via inhibition of the NF‐κB‐SREBP1 pathway.

## 4 Discussion

Aging is a major contributor to cognitive dysfunction, yet the mechanisms underlying age‐associated cognitive decline remain complex and incompletely understood. Among these, the role of senescent microglia has gained increasing attention but has not yet been fully elucidated. In the present study, we identified senescent microglia as a key driver of neuroinflammation and neuronal dysfunction in the aged brain. Importantly, we demonstrated that hUC‐MSCs alleviate cognitive decline by reducing LD accumulation and restoring functionality in senescent microglia, thereby mitigating neuroinflammation and delaying neuronal senescence. These findings underscore the potential of hUC‐MSCs as a promising therapeutic strategy for age‐related neurodegenerative diseases.

Microglia, as the resident immune cells of the central nervous system, are vital for maintaining neural homeostasis through surveillance, synaptic remodeling, and phagocytosis of apoptotic cells and debris. Under pathological conditions, microglia are activated, characterized by enlarged cell bodies and increased secretion of pro‐inflammatory factors (Kettenmann et al. 2011). Wyss‐Coray and colleagues reported a striking accumulation of lipid droplets in aging microglia in both murine and human brains, accompanied by impaired phagocytic capacity and a pro‐inflammatory profile, suggesting a dysfunctional state known as lipid droplet‐accumulating microglia (LDAM) (Marschallinger et al. 2020). In line with these findings, we observed microglial activation in the hippocampus of aged mice, indicated by increased cell number and soma size. Senescent microglia exhibited elevated pro‐inflammatory cytokine secretion and marked lipid droplet accumulation. TEM further revealed large LDs spatially adjacent to—but not enclosed within—lysosomes, suggesting impaired lysosome‐mediated lipophagy may contribute to LD buildup. Notably, hUC‐MSCs treatment reduced microglial LD accumulation in aged hippocampal tissues, and the remaining LDs were predominantly localized within lysosomes, implying that hUC‐MSCs may restore autophagic flux and lipophagy in senescent microglia.

LD accumulation is not only a result of impaired degradation but is also tightly regulated by biosynthetic pathways (Zadoorian et al. 2023). Transcriptomic analysis showed that hUC‐MSCs treatment downregulated the NF‐κB signaling pathway in aged hippocampal tissues, alongside reduced expression of sterol regulatory element‐binding protein 1 (SREBP1)—a transcription factor that orchestrates fatty acid and cholesterol biosynthesis (Oeckinghaus et al. 2011; He et al. 2024). Previous studies have demonstrated that NF‐κB can promote LD formation by upregulating SREBP1, particularly in hepatic and tumor cells (Yan and Horng 2020). Our findings support the notion that hUC‐MSCs reduce LD accumulation in senescent microglia via inhibition of the NF‐κB–SREBP1 axis, suggesting a mechanism of metabolic reprogramming that restores microglial function and limits inflammation.

Under physiological conditions, microglia indirectly regulate neuronal function by releasing cytokines, chemokines, and neuromodulators (Cserép et al. 2021). Under pathological conditions, however, activated microglia shift toward a pro‐inflammatory phenotype that exacerbates neuronal damage. In AD, for instance, activated microglia amplify Aβ deposition and tau hyperphosphorylation through IL‐1β‐mediated inflammatory signaling (Uddin and Lim 2022). This pathological shift is similarly evident in the aged brain, where senescent microglia create a microenvironment that is detrimental to neurogenesis (Zhang et al. 2021). Our results further revealed that senescent microglia not only amplify neuroinflammatory responses but also suppress apoptosis of aged neurons, potentially delaying the clearance of dysfunctional cells and accelerating neurodegeneration. Notably, we observed an abnormal accumulation of LDs in neurons co‐cultured with senescent microglia (data not shown), despite prior evidence indicating that neurons possess limited capacity for intrinsic LD synthesis and typically offload excess lipids to astrocytes for detoxification (Ioannou et al. 2019). These findings suggest that senescent microglia may disrupt this neuroprotective lipid‐transfer mechanism, possibly forcing neurons to retain LDs and thereby exacerbating lipotoxic stress through aberrant lipid droplet transfer. Collectively, our data indicate that senescent microglia contribute to neuronal damage via multiple pathways, including exacerbation of neuronal aging, inhibition of apoptosis, intensification of neuroinflammation, and enhancement of lipotoxic injury, which together contribute to cognitive dysfunction in the aging brain.

hUC‐MSCs are increasingly recognized in aging research due to their robust paracrine capacity, immunomodulatory effects, and regenerative potential (Wang et al. 2023; Wei et al. 2023). Previous studies have shown that hUC‐MSCs enhance muscle strength and function in mouse models of sarcopenia by promoting autophagy and reducing cellular senescence markers (Wang et al. 2023). In the central nervous system, hUC‐MSCs exert anti‐inflammatory effects by secreting immunoregulatory cytokines, thereby improving the brain's immune microenvironment and protecting against neuronal damage (Yang et al. 2023). Moreover, it has been demonstrated that hUC‐MSCs alleviate neuropathological changes in Alzheimer's disease (AD) models by secreting hepatocyte growth factor (HGF), enhancing synaptic plasticity, and promoting endogenous neurogenesis (Cao et al. 2017; Jia et al. 2020). In the present study, we demonstrated that hUC‐MSCs alleviated cognitive impairment in aged mice by rejuvenating microglial function and limiting lipid burden.

While these findings establish a phenotypic link between microglial senescence and cognitive decline, the mechanisms of action remain to be fully defined. Growing evidence points to extracellular vesicles (EVs), particularly exosomes, as principal mediators of MSC paracrine effects. For instance, hUC‐MSC‐derived EVs have been shown to improve skin photoaging by enhancing dermal hydration and collagen remodeling (Zhang et al. 2024), and Liu et al. demonstrated their ability to ameliorate Alzheimer's disease pathology via regulation of synaptic vesicle cycling (Li et al. 2024). In our study, hUC‐MSCs were co‐cultured with senescent microglia using a transwell system, which permits paracrine signaling without direct cell–cell contact. This setup suggests that soluble factors or EVs released by hUC‐MSCs may underlie the observed anti‐senescent effects. Future research should aim to isolate and characterize hUC‐MSC‐derived EVs to validate their functional contribution.

In conclusion, this study identifies a novel mechanism by which hUC‐MSCs ameliorate age‐associated cognitive impairment. Our findings demonstrate that hUC‐MSCs attenuate microglial senescence, reduce LD accumulation, and inhibit the NF‐κB‐SREBP1 signaling pathway, ultimately mitigating neuroinflammation and neuronal damage. While future studies (e.g., SREBP1/PLIN2 silencing) are needed to confirm a direct causal link between LD regulation and hUC‐MSC function, our findings highlight the therapeutic potential of hUC‐MSCs in age‐related neurodegenerative disorders.

Nonetheless, several limitations must be acknowledged. First, the precise molecular mechanisms by which the NF‐κB–SREBP1 axis regulates LD biogenesis in microglia require further investigation. Second, the contribution of senescent microglia to the accumulation of neuronal LD and the underlying mechanisms of lipid transfer are still poorly understood. Third, the exact paracrine signals or exosomal contents through which hUC‐MSCs suppress NF‐κB–SREBP1 activity require further investigation. Future studies addressing these questions will not only advance our understanding of microglial aging and neuroimmune dysregulation but may also lead to the development of targeted therapies for age‐related cognitive decline.

## Author Contributions

Lamei Cheng and Aihong Liang conceived the project and designed the experiments. Aihong Liang, Li Zhang, Yanan Li, Jing Peng, Yunduo Zhou, and Chao Yang performed the animal experiments; Jie Wang analyzed the transcriptome data; Hua Mei and Jun Zhu performed the hUC‐MSCs culture; Yizhong Yan and Li Zhang made the tissue sections; Aihong Liang and Li Zhang performed and analyzed LD accumulation in senescent microglia; Aihong Liang performed and analyzed the JSH‐23 and neuronal experiments; Aihong Liang wrote the original draft and review; Yu Zhou and Lamei Cheng revised and edited the manuscript; Na Xiao performed the animal experiments; Siqi Wang performed the hUC‐MSCs culture; Lamei Cheng supervised the study and provided funding.

## Conflicts of Interest

The authors declare no conflicts of interest.

## Supporting information


**Figure S1:** hUC‐MSCs ameliorate cognitive decline and hippocampal senescence in aged mice. (a) Schematics of mice treatment. (b) The mice total exploration times in NOR test. *n* = 6. (c)The percentage of SA‐β‐gal positive area in DG, CA1 and CA3 regions of the hippocampus was quantified by ImageJ software. *n* = 3 per group. (d) The protein levels of p16, p21 were quantified by Image J software. *n* = 4 per group. Data are presented as means ± SD. Statistical significance was determined by One‐way ANOVA, following normality and lognormality tests. ns, no significant, *p < 0.05, **p < 0.01.
**Figure S2:** hUC‐MSCs Attenuate Age‐Related Microglial Activation. (a) The number of IBA1^+^ cells were quantified by ImageJ software. *n* = 3 per group. (b) The size of IBA1^+^ cells body were quantified by ImageJ software. *n* = 3 per group. Data are presented as means ± SD. Statistical significance was determined by One‐way ANOVA, following normality and lognormality tests. ns, no significant, *p < 0.05, **p < 0.01.
**Figure S3:** The optimal concentration of H_2_O_2_ for inducing cellular senescence was determined. (a) Cell viability of BV2 cells in which treatment with different concentrations of H_2_O_2_ was determined using cck‐8. *n* = 6 per group. (b) Representative image of SA‐β‐gal staining in BV2 cells. (c) The protein levels of p21, γ‐H2AX were quantified by ImageJ software. *n* = 3 per group. (d) Representative image of BODIPY (green) in BV2 cells. (e) The protein level of PLIN2 was determined by western blot and quantified by ImageJ software. *n* = 3 per group. Data are presented as means ± SD. Statistical significance was determined by One‐way ANOVA, following normality and lognormality tests. ns, no significant, *p < 0.05.
**Figure S4:** JSH‐23 reduces lipid droplet accumulation in H_2_O_2_‐induced senescent microglia. (a) Representative image of oil red O staining in BV2 cells and (b) quantification of oil red O positive cells. *n* = 3 per group. (c) The mean fluorescence intensity of BODIPY was quantified by ImageJ software. *n* = 3 per group. Data are presented as means ± SD. Statistical significance was determined by One‐way ANOVA, following normality and lognormality tests. ns, no significant, *p < 0.05, **p < 0.01.

## Data Availability

The data that support the findings of this study are available on request from the corresponding author. The data are not publicly available due to privacy or ethical restrictions.
