# Supplementary material for: Human Umbilical Cord Mesenchymal Stem Cells Ameliorate Cognitive Decline by Restoring Senescent Microglial Function via NF‐κB‐SREBP1 Pathway Inhibition
Source: Aging Cell. 2025 Oct 13;24(12):e70259. doi: 10.1111/acel.70259 (PMC12686588; doi:10.1111/acel.70259)
Supplement: Supplementary file 1 — Figure S1: hUC‐MSCs ameliorate cognitive decline and hippocampal senescence in aged mice. (a) Schematics of mice treatment. (b) The mice total exploration times in NOR test. n = 6. (c)The percentage of SA‐β‐gal positive area in DG, CA1 and CA3 regions of the hippocampus was quantified by ImageJ software. n = 3 per group. (d) The protein levels of p16, p21 were quantified by Image J software. n = 4 per group. Data are presented as means ± SD. Statistical significance was determined by One‐way ANOVA, following normality and lognormality tests. ns, no significant, *p < 0.05, **p < 0.01. Figure S2: hUC‐MSCs Attenuate Age‐Related Microglial Activation. (a) The number of IBA1+ cells were quantified by ImageJ software. n = 3 per group. (b) The size of IBA1+ cells body were quantified by ImageJ software. n = 3 per group. Data are presented as means ± SD. Statistical significance was determined by One‐way ANOVA, following normality and lognormality tests. ns, no significant, *p < 0.05, **p < 0.01. Figure S3: The optimal concentration of H2O2 for inducing cellular senescence was determined. (a) Cell viability of BV2 cells in which treatment with different concentrations of H2O2 was determined using cck‐8. n = 6 per group. (b) Representative image of SA‐β‐gal staining in BV2 cells. (c) The protein levels of p21, γ‐H2AX were quantified by ImageJ software. n = 3 per group. (d) Representative image of BODIPY (green) in BV2 cells. (e) The protein level of PLIN2 was determined by western blot and quantified by ImageJ software. n = 3 per group. Data are presented as means ± SD. Statistical significance was determined by One‐way ANOVA, following normality and lognormality tests. ns, no significant, *p < 0.05. Figure S4: JSH‐23 reduces lipid droplet accumulation in H2O2‐induced senescent microglia. (a) Representative image of oil red O staining in BV2 cells and (b) quantification of oil red O positive cells. n = 3 per group. (c) The mean fluorescence intensity of BODIPY was quantified [file ACEL-24-e70259-s001.docx]

**Supplement**

**Human umbilical cord-derived mesenchymal stem cells ameliorate cognitive dysfunction in aging mice and reduce lipid droplet accumulation in senescent microglia via paracrine**

Aihong Liang^1,2,4#^, Li Zhang^1,4#^, Jing Peng^2,3^, Yanan Li^1,4^, Yunduo Zhou^2,3,6^, Chao Yang^2,3^, Jie Wang^2,3^, Yizhong Yan^2,3^, Hua Mei^2,3^, Jun Zhu^2,6^, Siqi Wang^2,3^, Na Xiao^2,3,6^, Yu Zhou^2,3,5^, Lamei Cheng^1,2,3,4,6^*

1 Institute of Reproductive and Stem Cell Engineering, Xiangya School of Basic Medical Science, Central South University, Changsha, China

2 National Engineering Research Center of Human Stem Cells, Changsha, China

3 Hunan Guangxiu Hi-tech Life Technology Co. Ltd, Changsha, China

4 NHC Key Laboratory of Human Stem Cell and Reproductive Engineering, Xiangya School of Basic Medical Science, Central South University, Changsha, China

5 Department of Neurosurgery, The Second Xiangya Hospital of Central South University, Changsha, China

6 Guangxiu Hospital, Hunan Normal University, Changsha, China

**Supplementary Figure 1**

**
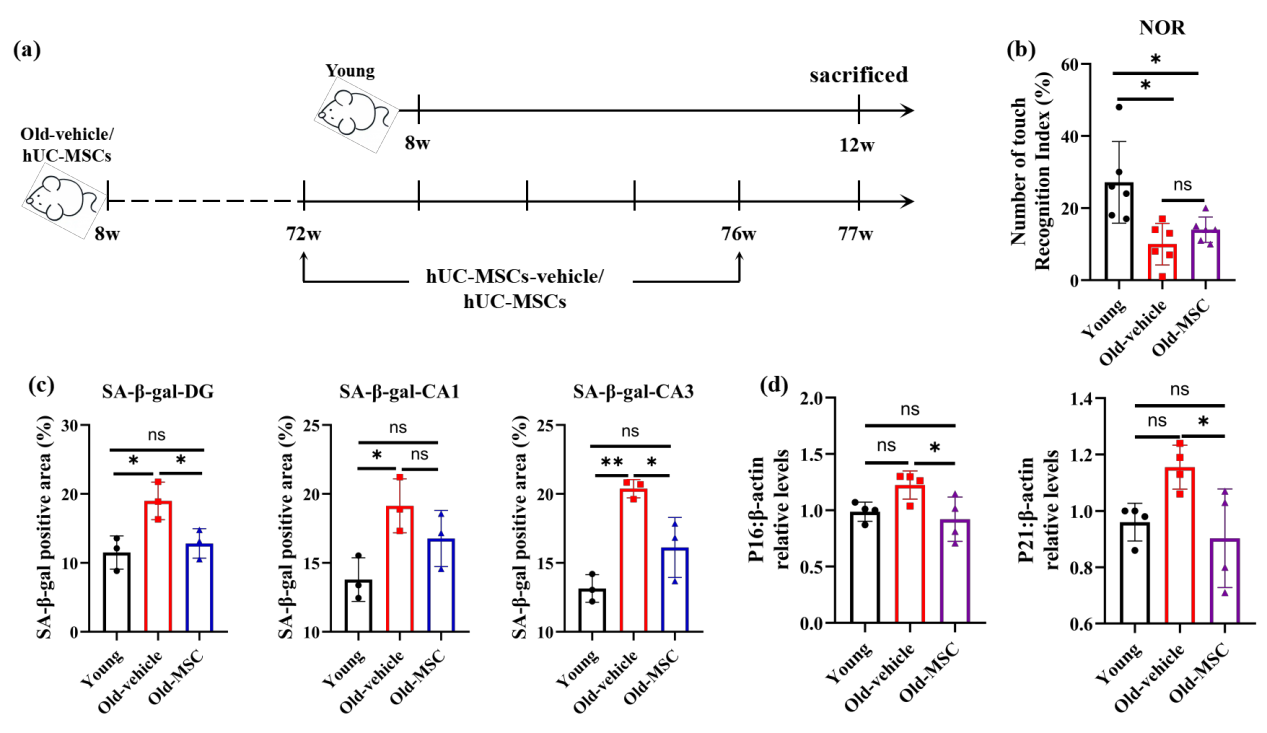
**

Supplementary Figure1 hUC-MSCs ameliorate cognitive decline and hippocampal senescence in aged mice. (a) Schematics of mice treatment. (b) The mice total exploration times in NOR test. n=6. (c)The percentage of SA-β-gal positive area in DG, CA1 and CA3 regions of the hippocampus was quantified by ImageJ software. n=3per group. (d) The protein levels of p16, p21 were quantified by Image J software. n=4 per group. Data are presented as means ± SD. Statistical significance was determined by One-way ANOVA, following normality and lognormality tests. ns, no significant, **p*< 0.05, ***p*< 0.01.

**Supplementary Figure 2**

**
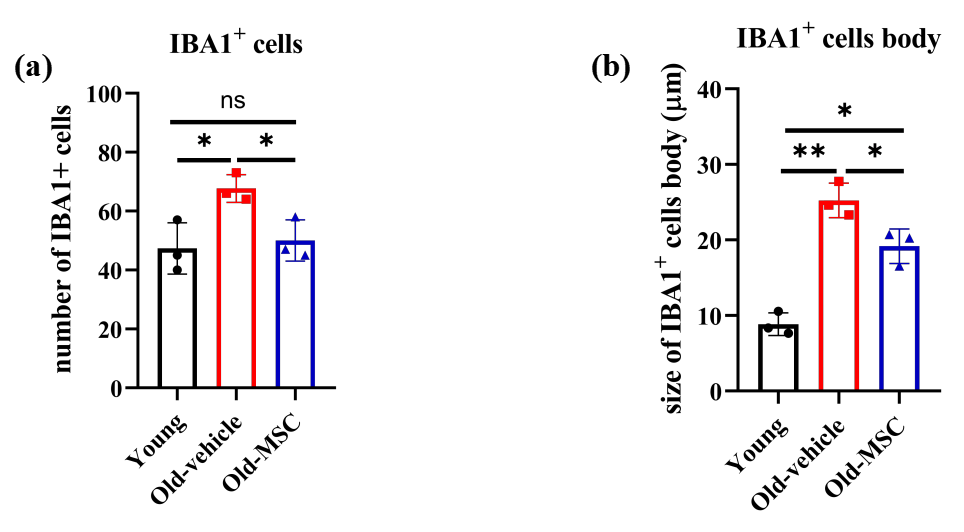
**

Supplementary Figure 2 hUC-MSCs Attenuate Age-Related Microglial Activation.

(a) The number of IBA1^+^ cells were quantified by ImageJ software. n=3 per group. (b) The size of IBA1^+^ cells body were quantified by ImageJ software. n=3 per group. Data are presented as means ± SD. Statistical significance was determined by One-way ANOVA, following normality and lognormality tests. ns, no significant, **p*< 0.05, ***p*< 0.01.

**
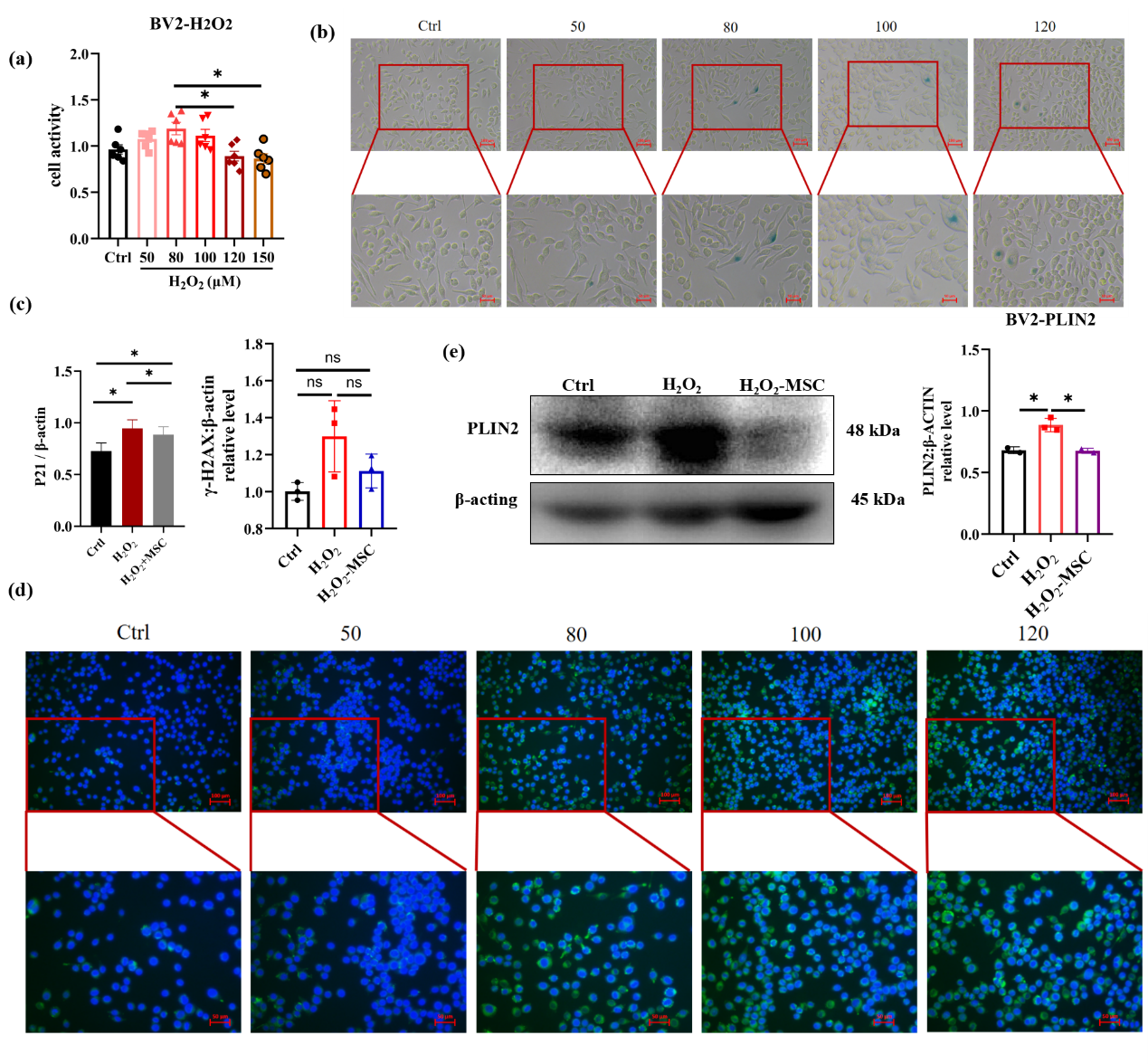
**

**Supplementary Figure 3**

Supplementary Figure 3 The optimal concentration of H_2_O_2_ for inducing cellular senescence was determined. (a) Cell viability of BV2 cells in which treatment with different concentrations of H_2_O_2_ was determined using cck-8. n=6 per group. (b) Representative image of SA-β-gal staining in BV2 cells. (c) The protein levels of p21, γ-H2AX were quantified by ImageJ software. n=3 per group. (d) Representative image of BODIPY (green) in BV2 cells. (e) The protein level of PLIN2 was determined by western blot and quantified by ImageJ software. n=3 per group. Data are presented as means ± SD. Statistical significance was determined by One-way ANOVA, following normality and lognormality tests. ns, no significant, **p*< 0.05.

**Supplementary Figure 4**

**
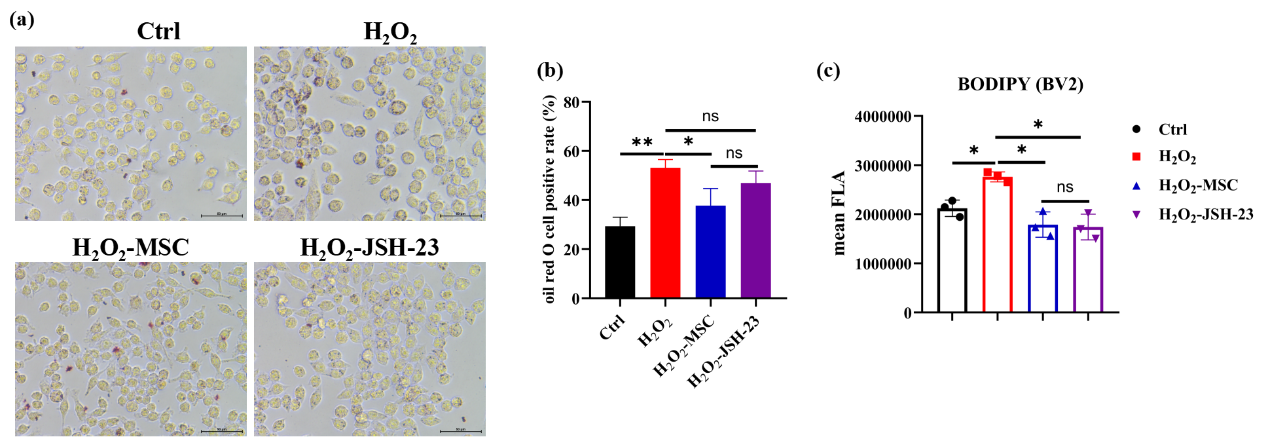
**

Supplementary Figure 4 JSH-23 reduces lipid droplet accumulation in H_2_O_2_-induced senescent microglia. (a) Representative image of oil red O staining in BV2 cells and (b) quantification of oil red O positive cells. n=3 per group. (c) The mean fluorescence intensity of BODIPY was quantified by ImageJ software. n=3 per group. Data are presented as means ± SD. Statistical significance was determined by One-way ANOVA, following normality and lognormality tests. ns, no significant, **p*< 0.05, ***p*< 0.01.
